# Consequences of continuing renin angiotensin aldosterone system antagonists in the preoperative period: a systematic review and meta-analysis

**DOI:** 10.1186/s12871-018-0487-7

**Published:** 2018-02-26

**Authors:** Qiong Ling, Yu Gu, Jiaxin Chen, Yansheng Chen, Yongyong Shi, Gaofeng Zhao, Qianqian Zhu

**Affiliations:** 1 Department of Anesthesiology, The Second Affiliated Hospital of Guangzhou University of Chinese Medicine, Guangzhou City, 510120 People’s Republic of China; 2 Department of Anesthesiology, The Third Affiliated Hospital of Sun Yat-Sen University, Guangzhou City, Guangdong Province 510630 People’s Republic of China

**Keywords:** Angiotensin-converting enzyme inhibitors (ACEIs), Angiotensin II receptor blockers (ARBs), Intraoperative hypotension

## Abstract

**Background:**

Patients who use angiotensin-converting enzyme inhibitors (ACEIs)/angiotensin II receptor blockers (ARBs) are prone to developing side effects like hypotension and even refractory hypotension during anesthesia use, and whether ACEIs/ARBs should be continued or discontinued in such patients remains debatable. The present systematic review and meta-analysis was conducted to clarify the consequences of continuing or withholding these drugs, especially with regards to the incidence of intraoperative hypotension, in patients who continue to use ACEIs/ARBs on the day of their scheduled surgery.

**Methods:**

Studies with data pertinent to the incidence of intraoperative hypotension during anesthesia use in patients who continued the use of ACEIs/ARBs on the day of their scheduled surgery were considered for inclusion.

**Results:**

Thirteen studies reporting on the incidences of intraoperative hypotension between patients who continued receiving ACEIs/ARBs and those who did not on the day of their surgical procedure were included. The pooled effects showed that hypotension during anesthesia was more likely to develop in patients who continued to take ACEIs/ARBs when compared to those who did not (RR = 1.41, 95% CI: 1.21–1.64). However, there were no significant differences between these groups of patients with regards to postoperative complications including ST-T abnormalities, myocardial injury, myocardial infarction, stroke, major adverse cardiac events, acute kidney injury, or death (RR = 1.25, 95% CI: 0.76–2.04). The differences remained similar in subgroup analyses and sensitivity analyses.

**Conclusions:**

No sufficient available evidence to recommend discontinuing ACEIs/ARBs on the day of surgery was found in this literature review and meta-analysis. However, anesthetists should be cautious about the risk for intraoperative hypotension in patients chronically receiving ACEIs/ARBs, and should know how to treat it effectively.

## Background

An increasing number of patients who have hypertension and chronic heart diseases continue to be scheduled for elective surgery. However, for those patients who use angiotensin-converting enzyme inhibitors (ACEIs)/angiotensin II receptor blockers (ARBs), the question of whether these medications should be continued or discontinued on the day of surgery remains under discussion [1]. ACEIs/ARBs are not only widely used as antihypertensive medications, but also for treating chronic heart diseases or other diseases, such as diabetic nephropathy [2]. Furthermore, the use of ACEIs/ARBs has shown various beneficial effects [3, 4].

However, patients using ACEIs/ARBs have been reported to be prone to side effects like hypotension and even refractory hypotension during the administration of anesthesia [5, 6]. Therefore, some anesthetists have suggested the possibility of discontinuing these drugs in order to maintain the patient’s hemodynamic stability during surgery [7, 8]. However, other research has shown that the discontinuation of ACEIs/ARBs preoperatively did not decrease the incidence of hypotension, and that the recommendation of discontinuing ACEIs/ARBs should be taken with reservations [9, 10]. One study indicated that patients who discontinued these drugs on the day of surgery required more vasodilators to control hypertension after surgery [9]. Regardless of the predominant opinion, it is clear that the present concerns regarding continuing or discontinuing these drugs before surgery need to be explored. Besides being first-line antihypertensive drugs, these drugs also decrease morbidity and mortality, and prevent secondary strokes in patients with chronic heart diseases [11].

In order to clarify the consequences of continuing patients on ACEIs/ARBs on the day of their scheduled surgery, the present systematic review and meta-analysis was conducted.

## Methods

### Search strategy

The following electronic databases were searched: the Cochrane Library, PubMed, the Web of Knowledge, and Elsevier (ScienceDirect OnLine, SDOL) to retrieve studies investigating the incidence of hypotension in those patients continuously receiving ACEIs/ARBs, especially in patients continuing their use of ACEIs/ARBs on the day of their surgery.

Text headings and medical subject heading (MeSH) terms for the search included “angiotensin-converting enzyme inhibitors/ACEIs,” “angiotensin II receptor blockers/ARBs,” “angiotensin receptor antagonist,” “renin-angiotensin system (RAS)/RAS inhibitor,” “renin-angiotensin aldosterone system (RAAS)/RAAS inhibitor,” “hypotension,” and “low blood pressure.”

The search strategy included considering any terms pertinent to renin-angiotensin system/RAS inhibitors and any terms related to hypotension. Eligible trials were identified via electronic searches from 1981, when captopril, the first ACEI, was approved by the United States Food and Drug Administration, up to July 1, 2017. A hand-search method was used to examine the reference lists of some of the identified trials. The Preferred Reporting Items for Systematic Reviews and Meta-Analyses (PRISMA) guidelines were used to guide the meta-analysis.

### Inclusion and exclusion criteria

Case-control studies, cohort studies, and/or randomized controlled trials were considered for inclusion if they met the following criteria: (i) written in the English language; (ii) enrolled adults scheduled for elective or emergency surgery who were chronically using ACEIs/ARBs; and (iii) compared the incidence of hypotension during anesthesia in patients continuing to receive ACEIs/ARBs with those who did not receive these drugs on the day of surgery. The exclusion criteria were: (i) formatted as a letter, review or meeting abstract; (ii) containing a lack of hypotension data as incidences or numbers; and (iii) containing no delineation between patients continuing to receive ACEIs/ARBs and those who were not, or those who were using other drugs or who had never used antihypertensive drugs. Only published data were included in the present study.

### Data extraction

The data were extracted independently by three reviewers (Qiong Ling, Yu Gu and Jiaxin Chen) and validated by a fourth one (Qianqian Zhu). The following information was extracted from each included study: the name of the primary author, year of publication, geographical location, number of participants, type of surgery, and anesthesia.

### Statistical analysis

The meta-analysis was performed in Review Manager 5 (The Cochrane Collaboration, Oxford, England). The pooled effect of continuing ACEI/ARB use on the incidence of hypotension during anesthesia use was demonstrated as relative risk (RR) with a 95% confidence interval (CI). Subgroup analyses were used according to whether the study included patients chronically taking ACEIs/ARBs or not. Sensitivity analyses according to the type of included studies were also used. A chi-squared test was used to assess the heterogeneity. An I^2^ value of less than 25% was regarded as representing no heterogeneity. A random effects model was used when heterogeneity was found to exist among the studies analyzed. Begg’s and Egger’s tests were used to assess the publication bias, and were performed using Stata 12.1 (Stata Corp, College Station, TX, USA).

## Results

### Characteristics of the included studies

**Fig. 1:**
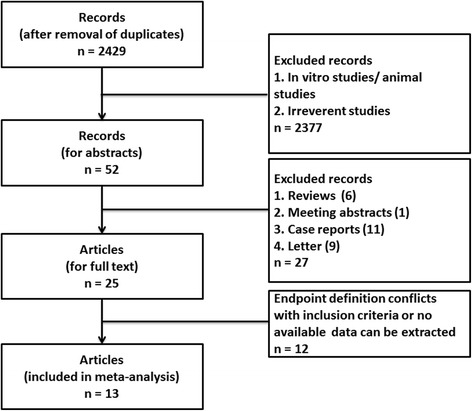
The flow chart of inclusion and exclusion

The search strategy yielded 2,429 non-duplicated entries. Thirteen studies were included in the final analysis [7, 9, 12–22]. The inclusion and exclusion processes of the studies are shown in Fig. 1.

Seven studies investigated the hypotension differences in patients who ceased taking ACEIs/ARBs on the day of surgery and those who did not. Six studies compared the incidences of hypotension between patients who continued receiving ACEIs/ARBs on the day of surgery and those who did not receive these drugs chronically. The details of the included studies are shown in Table 1. The quality of observational studies was assessed with the use of the Newcastle–Ottawa Scale (NOS) [23] (Table 1).

**Table 1:** Characteristics of studies included in meta-analysis

Author, year	Country	Type of surgery	Type of anesthesia	Number of participants	Female (%)	Age (Mean)	Postoperative complications	Definition of hypotension	Type of study	Quality score^i^
Bertrand, 2001 [7]	France	Elective major vascular sugery	General	37	12.2	68	ST-T abnormalities	SBP^a^< 80 mmHg	RCT	RCT
Brabant, 1999 [12]	France	Elective vascular surgery	General	39	16.7	69	MI^b^	SBP < 90 mmHg or 30% baseline	Prospective observational	8
Calloway, 2014 [13]	USA	Total knee arthroplasty	Neuraxial	60	61	66	MI, stroke/TIA^c^, AKI^d^, death	SBP ≤85 mmHg	Retrospective observational	8
Colson, 1992 [18]	France	Coronary artery bypass graft surgery	General	16	12.5	65	N/A^e^	MAP^f^ < 70 mmHg	Prospective observational	6
Comfere, 2005 [15]	USA	Non-cardiovascular	General	267	44	66(Median)	MI, stroke, AKI, death	SBP ≤85 mmHg	Retrospective observational	9
Coriat, 1994 [16]	France	Peripheral vascular	General	51	N/A	67	N/A	SBP < 90 mmHg	RCT^g^	RCT
Licker, 2000 [19]	Switzerland	Coronary artery bypass graft	General	32	22	60	N/A	SBP < 90 mmHg	Prospective observational	7
Pigott, 1999 [9]	UK	Coronary artery bypass graft	General	40	12.5	63	N/A	SBP < 85 mmHg	RCT	RCT
Roshanov, 2017 [20]	Eight countries	Noncardiac Surgery	NA	4802	N/A	N/A	myocardial injury, stroke, death	SBP < 90 mmHg	Prospective observational	8
Ryckwaert, 1997 [14]	France	Coronary artery bypass graft	General	18	11	62	N/A	70mmHg	Prospective observational	7
Salvetti, 2016 [17]	Italy	Elective bariatric surgery	General	26	73%	47	N/A	N/A	Prospective observational	8
Steely, 2016 [21]	New England	Carotid endarterectomy	General/ Neuraxial	2878	N/A	N/A	MACE^h^, stroke/ death	N/A	Retrospective observational	9
Zainudheen, 2017 [22]	Woolloongabba	Elective orthopaedic surgery	General/ Neuraxial	258	58.5%	69.8	MACE, AKI	SBP < 90 mmHg	Retrospective observational	7

^a^, SBP, systolic blood pressure; ^b^, MI, myocardial infarction; ^c^, TIA, transient ischemic attack; ^d^, AKI, acute kidney injury; ^e^, N/A, not available;^f^, mean arterial pressure; ^g^, RCTs, randomized controlled trials; ^h^, MACE, major adverse cardiac event

^i^the quality score was assessed with the use of the Newcastle–Ottawa Scale (NOS)

### Meta-analyses

**Fig. 2:**
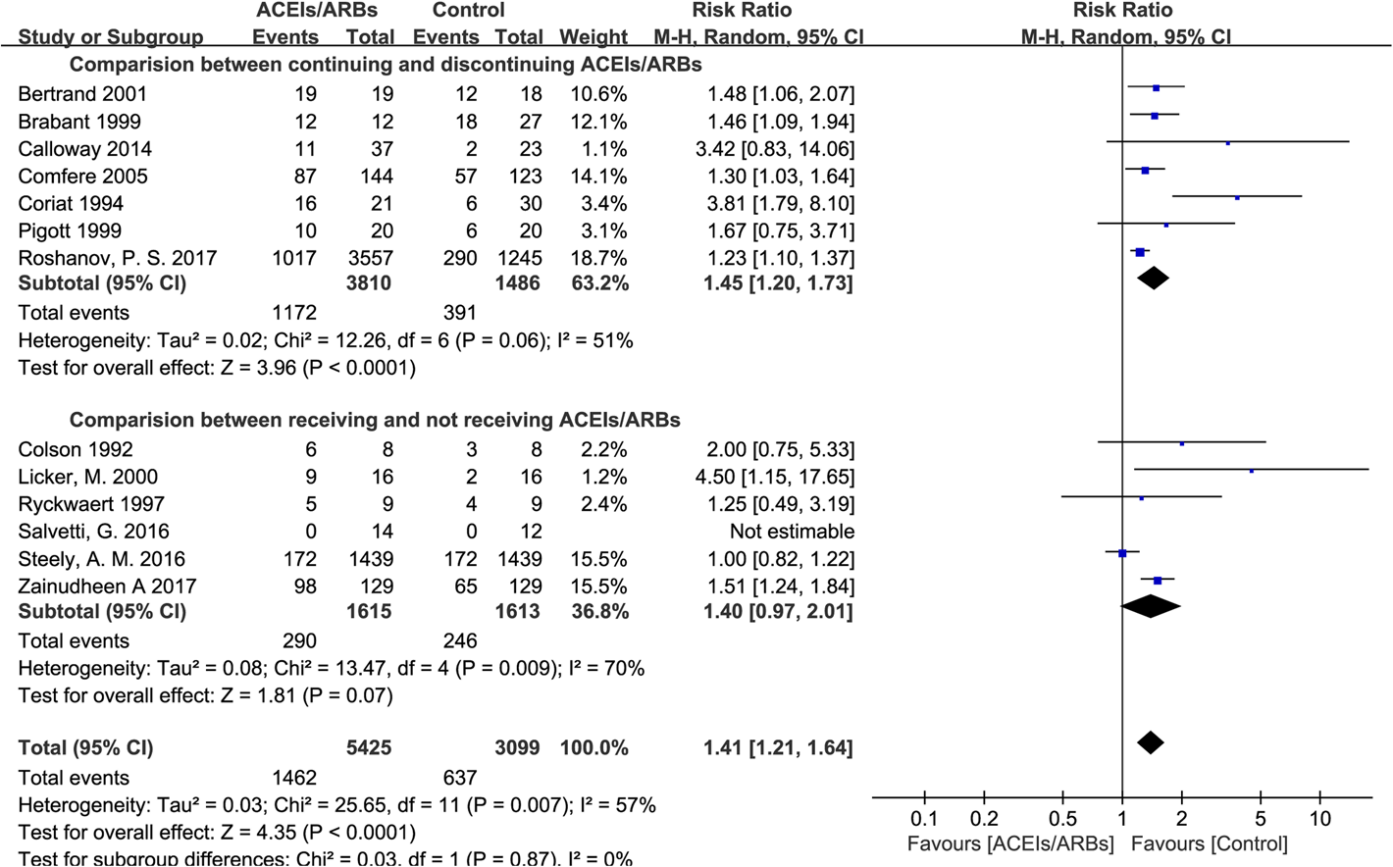
Intraoperative hypotension between patients continuing and not receiving ACEIs/ARBs

Pooled effects showed that patients who continued taking ACEIs/ARBs on the day of surgery were more like to develop hypotension during anesthesia use (RR = 1.41, 95% CI: 1.21–1.64; Fig. 2), in comparison with those who were not receiving the drugs. The subgroup analysis demonstrated that, in comparison with patients who continued taking ACEIs/ARBs on the day of surgery, hypotension during anesthesia was more prone to occur in patients who ceased taking the drugs prior to surgery (RR = 1.45, 95% CI: 1.20–1.73; Fig. 2), but not in those who did not receive the drugs at baseline (RR = 1.40, 95% CI: 0.97–2.01; Fig. 2). The details of the heterogeneities between the studies are showed in Fig. 2.

However, notably, the definitions of hypotension were different in the different studies (Table 1).

### Postoperative complication

**Fig. 3:**
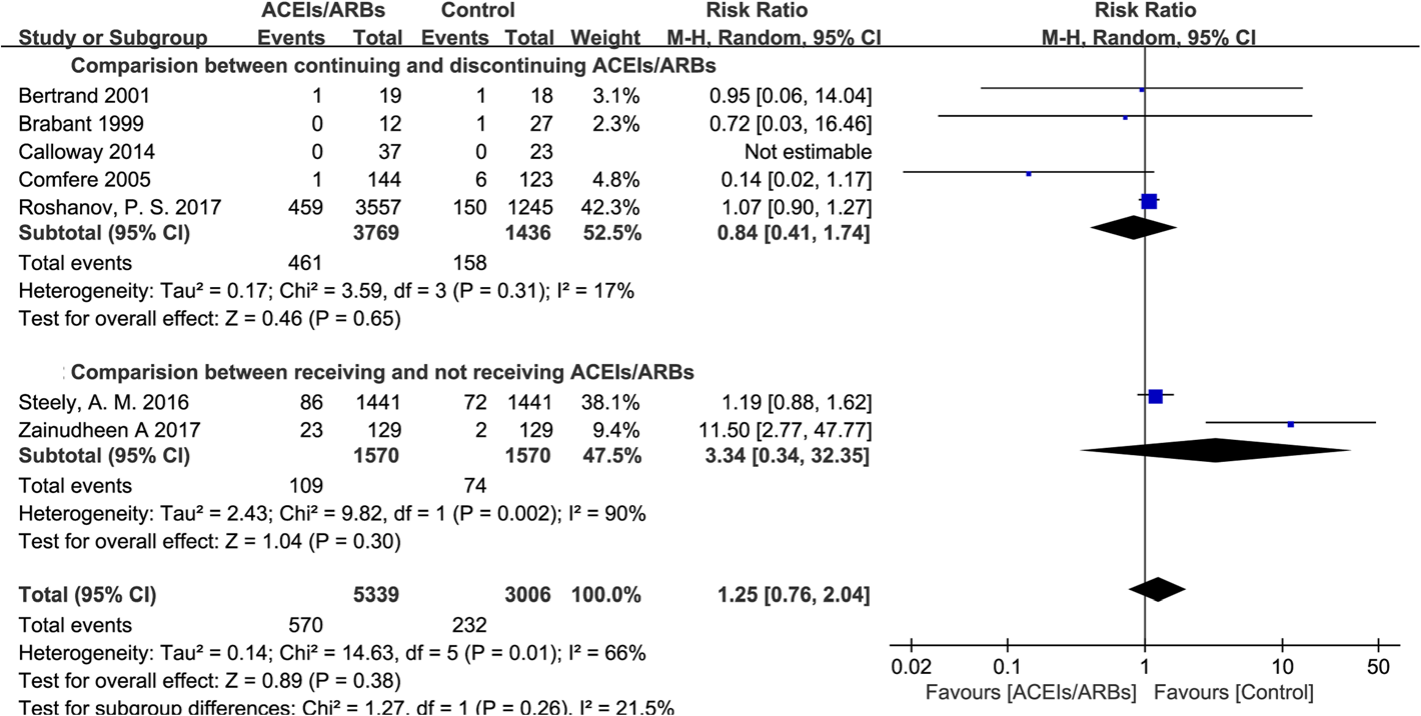
Postoperative complications between patients continuing and not receiving ACEIs/ARBs

Seven studies reported postoperative complications, including ST-T abnormalities, myocardial injury, myocardial infarction, stroke, major adverse cardiac events, acute kidney injury, or death (Table 1) [7, 12, 13, 15, 20–22]. There were no significant differences between patients who continued receiving ACEIs/ARBs and those who did not with regards to the postoperative complications mentioned above (RR = 1.25, 95% CI: 0.76–2.04; Fig. 3). The differences remained similar in the subgroup analyses (Fig. 3).

**Fig. 4:**
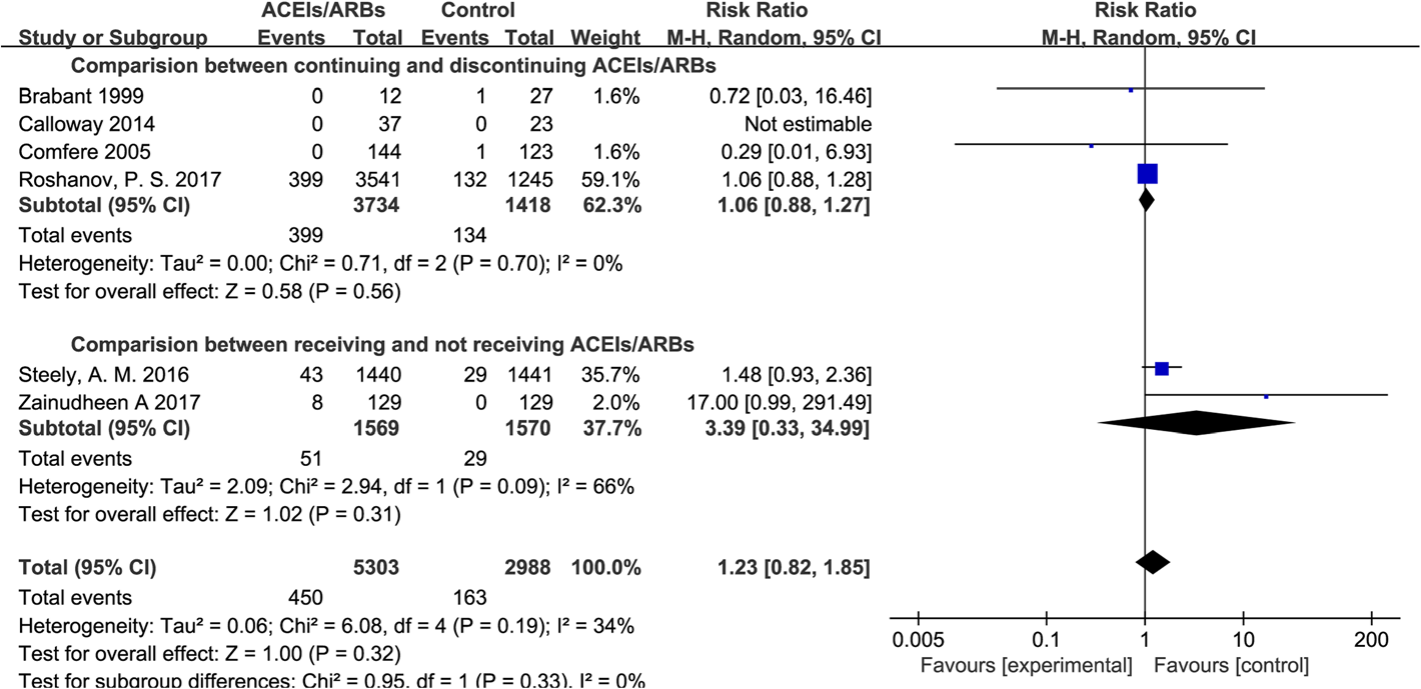
Cardiac complications between patients continuing and not receiving ACEIs/ARBs

It was observed that there were no significant differences in the prevalence of cardiac complications, including myocardial injury, myocardial infarctions, and major adverse cardiac events, between the patients who continued receiving ACEIs/ARBs and those who did not (RR = 1.23, 95% CI: 0.82–1.85; Fig. 4).

### Subgroup and sensitivity analyses

The results of subgroup and sensitivity analyses are list in Table 2.

**Table 2:** Results of subgroup and sensitivity analyses

	RR and I^b^ for hypotension	RR and I^b^ all complications	RR and I^b^ all cardiac complications
Subgroup analyses			
Results of orthopaedic surgery surgery	1.70 (0.95–3.04), 26%	N/A^a^	N/A
Results of cardiac surgery	2.00(1.23–3.26), 0%	N/A	N/A
Results of noncardiac surgery	1.37(1.17–1.61), 67%	1.25(0.76–2.04), 66%	1.23(0.82–1.85), 34%
Results of vascular surgery	1.51(1.02–2.23), 82%	1.18(0.88–1.60), 0%	1.46(0.92–2.31), 0%
Sensitivity analyses			
Results of RCTs^b^	2.01(1.07–3.77), 68%	N/A	N/A
Results of prospective observational studies	1.35(1.11–1.66), 26%	1.07(0.90–1.27), 0%	1.06(0.88–1.28), 0%
Results of retrospective observational studies	1.29(0.99–1.69), 73%	1.42(0.22–9.24), 85%	1.87(0.35–10.06), 85%

^a^, *NA* not available, ^b^, *RCTs* randomized controlled trials

It was observed that there were no significant differences in the prevalence of postoperative complications or cardiac complications, between the patients who continued receiving ACEIs/ARBs and those who did not in all subgroup and sensitivity analyses.

### Publication bias

Begg’s and Egger’s tests were used to assess the publication bias for all the included studies. No significant publication bias was found (*p* > 0.05 for both tests).

## Discussion

Based on the available data, the present systematic review and meta-analysis of 13 studies demonstrated that patients who continued taking ACEIs/ARBs on the day of their surgery were more likely than those who did not, to develop hypotension during anesthesia. However, receiving ACEIs/ARBs on the day of surgery did not increase the incidences of noted postoperative complications, including myocardial infarction, stroke, acute kidney injury, and death. The subgroup and sensitivity analyses showed that the association is similar only when comparing the patients who ceased taking ACEIs/ARBs prior to surgery with those who continued taking the drugs.

RAAS antagonists or ACEIs/ARBs, are the first-line drugs for the treatment of hypertension and chronic heart failure. Because intraoperative hemodynamic instability, especially refractory hypotension, has been observed in patients who have been treated chronically with ACEIs/ARBs [24–26], some researchers have suggested discontinuing these drugs on the day of surgery [7, 8]. RAAS antagonists play a major role in regulating and maintaining normal blood pressure, especially during general anesthesia use [27]^.^ Additionally, some researchers have suggested that ACEIs/ARBs reduce the adrenergic vasoconstrictive response [19]. This might partly explain why ACEI/ARB-associated hypotension was refractory and resistant to phenylephrine, ephedrine, and norepinephrine [6, 28]. However, severe or refractory hypotension during anesthesia administration in patients chronically receiving ACEIs/ARBs has only been reported in several cases [24, 25]. In most cases, hypotension was sensitive to intravenous fluid infusion and vasoconstrictors, and continuing ACEIs/ARBs on the day of surgery did not increase the incidence of severe or refractory hypotension. Terlipressin is known to be effective in rapidly correcting refractory hypotension, even after the failure of ephedrine in patients chronically treated with ACEIs/ARBs, without impairing left ventricular function [29, 30].

The most concerning factor of hypotension is the occurrence of ischemia-related events, including myocardial injury, myocardial infarction, stroke, and acute kidney injury. However, the results of the present study showed that continuing ACEIs/ARBs on the day of surgery did not increase the incidence of postoperative complications such as myocardial injury, myocardial infarction, stroke, acute kidney injury, or death. In line with the present study, another recent study conducted in eight countries also demonstrated that intraoperative hypotension was not significantly associated with the composite outcome of death, myocardial injury, or stroke within the 30 days after surgery [20]. Furthermore, several myocardial infarctions were reported in patients who discontinued the use of ACEIs/ARBs, though a previous meta-analysis showed that there was no more risk of postoperative myocardial infarction in patients continuing than in those discontinuing ACEIs/ARBs preoperatively [31]. ACEIs/ARBs may protect patients from myocardial infarction, cardiovascular mortality, and morbidity, which might be attributed to the ability of these drugs to prevent ventricular remodeling and improving left ventricular function [3, 32]. A previous study suggested an association between uninterrupted reception of ACEIs/ARBs and a reduction in ischemia-related myocardial cell injury in cardiac surgery [33]. Therefore, some experts recommend that these drugs should not be discontinued before surgery [34]. Furthermore, treatment with ACEIs/ARBs after acute myocardial infarction was associated with improved long-term survival and low rates of adverse renal events [35].

To date, there has been no large randomized controlled trial (RCT) that explores the long-term effects of discontinuing ACEIs/ARBs. Furthermore, no serious outcomes have occurred, though some intraoperative refractory hypotension cases were reported in patients continuing the use of ACEIs/ARBs on the day of surgery in the current literature. Refractory hypotension could be treated efficiently by a combination of fluid infusion and vasoconstrictors, especially terlipressin [36]. Therefore, in order to avoid a drug holiday and forgetting to restart using the drugs, physicians should be cautious when recommending that these drugs be discontinued.

### Strengths and limitations

There are some limitations in the present meta-analysis. First, the included studies varied in design and quality, and no RCT was identified that involved a large number of participants. Though a recent study conducted in eight countries involved many patients, it was an observational study [20]. Many potential confounders might limit the statistical power of the results of that study. Secondly, the definition of hypotension or refractory hypotension was not consistent in each study, which might lead to bias of the results. Furthermore, there were no data on the long-term effects of continuing or discontinuing ACEIs/ARBs on the day of surgery, though several studies reported the occurrence of myocardial infarction based on troponin levels and electrocardiogram findings in a short time after surgery, and one study reported postoperative death [12, 16]. Therefore, the above mentioned factors might weaken the results of the present study.

## Conclusion

In conclusion, the data available at the time this study was conducted did not provide sufficient evidence to support that continuing ACEIs/ARBs on the day of surgery in patients who chronically received these drugs is associated with obvious disadvantages. No significant incidences of severe or refractory hypotension or postoperative ischemia-related complications were observed between those patients continuing ACEIs/ARBs and those who either discontinued these drugs or continued using other antihypertensive drugs. Therefore, evidence supporting the discontinuation of ACEIs/ARBs on the day of surgery is lacking. However, anesthetists should be cautious about the possibility of hypotension in patients chronically receiving ACEIs/ARBs, and should know how to treat it effectively. To explore the long-term effects of continuing or discontinuing ACEIs/ARBs in patients, RCTs involving a large number of patients are required.

## Abbreviations

ACEIs: Angiotensin-converting enzyme inhibitors;
ARBs: Angiotensin II receptor blockers
CI: Confidence interval
RAAS: Renin-angiotensin aldosterone system
RAS: Renin-angiotensin system
RR: Relative risk
